# Development and Validation of a Novel Gene Signature for Predicting the Prognosis of Idiopathic Pulmonary Fibrosis Based on Three Epithelial-Mesenchymal Transition and Immune-Related Genes

**DOI:** 10.3389/fgene.2022.865052

**Published:** 2022-04-26

**Authors:** Jiafeng Zheng, Hanquan Dong, Tongqiang Zhang, Jing Ning, Yongsheng Xu, Chunquan Cai

**Affiliations:** ^1^ Department of Pediatric Respiratory Medicine, Tianjin Children’s Hospital (Tianjin University Children’s Hospital), Tianjin, China; ^2^ Tianjin Institute of Pediatrics(Tianjin Key Laboratory of Birth Defects for Prevention and Treatment), Tianjin Children’s Hospital (Tianjin University Children’s Hospital), Tianjin, China

**Keywords:** idiopathic pulmonary fibrosis, epithelial–mesenchymal transition, immune, prognosis, marker

## Abstract

**Background:** Increasing evidence has revealed that epithelial–mesenchymal transition (EMT) and immunity play key roles in idiopathic pulmonary fibrosis (IPF). However, correlation between EMT and immune response and the prognostic significance of EMT in IPF remains unclear.

**Methods:** Two microarray expression profiling datasets (GSE70866 and GSE28221) were downloaded from the Gene Expression Omnibus (GEO) database. EMT- and immune-related genes were identified by gene set variation analysis (GSVA) and the Estimation of STromal and Immune cells in MAlignant Tumors using Expression data (ESTIMATE) algorithm. Gene Ontology (GO) and Kyoto Encyclopedia of Genes and Genomes (KEGG) pathway enrichment analyses were performed to investigate the functions of these EMT- and immune-related genes. Cox and least absolute shrinkage and selection operator (LASSO) regression analyses were used to screen prognostic genes and establish a gene signature. Gene Set Enrichment Analysis (GSEA) and Cell-type Identification By Estimating Relative Subsets Of RNA Transcripts (CIBERSORT) were used to investigate the function of the EMT- and immune-related signatures and correlation between the EMT- and immune-related signatures and immune cell infiltration. Quantitative real-time polymerase chain reaction (qRT-PCR) was performed to investigate the mRNA expression of genes in the EMT- and immune-related signatures.

**Results:** Functional enrichment analysis suggested that these genes were mainly involved in immune response. Moreover, the EMT- and immune-related signatures were constructed based on three EMT- and immune-related genes (IL1R2, S100A12, and CCL8), and the K–M and ROC curves presented that the signature could affect the prognosis of IPF patients and could predict the 1-, 2-, and 3-year survival well. Furthermore, a nomogram was developed based on the expression of IL1R2, S100A12, and CCL8, and the calibration curve showed that the nomogram could visually and accurately predict the 1-, 2-, 3-year survival of IPF patients. Finally, we further found that immune-related pathways were activated in the high-risk group of patients, and the EMT- and immune-related signatures were associated with NK cells activated, macrophages M0, dendritic cells resting, mast cells resting, and mast cells activated. qRT-PCR suggested that the mRNA expression of IL1R2, S100A12, and CCL8 was upregulated in whole blood of IPF patients compared with normal samples.

**Conclusion:** IL1R2, S100A12, and CCL8 might play key roles in IPF by regulating immune response and could be used as prognostic biomarkers of IPF.

## Introduction

Idiopathic pulmonary fibrosis (IPF) is a chronic interstitial lung disease of unknown etiology. IPF is the most common type of idiopathic interstitial pneumonia (IIP), accounting for 25–30% of patients diagnosed with interstitial lung disease (Richeldi et al., 2017; Lederer and Martinez, 2018). IPF is characterized by clinical symptoms of cough and dyspnea, limited lung function, impaired gas exchange, and progressive pulmonary scarring (Martinez et al., 2017; Richeldi et al., 2017; Lederer and Martinez, 2018; Sgalla et al., 2018; Spagnolo et al., 2021). The incidence, disability, and mortality of IPF are high; the annual incidence is 3–18 million in North America and Europe, and it is increasing year by year (Ley and Collard, 2013; Hutchinson et al., 2015). Although the disease process varies greatly among individuals, growing studies have found that the prognosis of IPF patients is poor, with a median survival time of only 3–5 years from the time of diagnosis (Raghu et al., 2006; Fernández Pérez et al., 2010; Martinez et al., 2017). Unfortunately, the treatment options for IPF patients are still limited. Lung transplantation remains the only treatment, which significantly improves survival in carefully selected patients. After more than a decade of unsuccessful clinical trials, in 2014, the first two drugs for the treatment of IPF were approved by the FDA in the United States, namely, pirfenidone and nindanib. The advent of these two drugs changed the situation that there was no cure for IPF and brought a dawn for the treatment of IPF (King et al., 2014; Richeldi et al., 2014). However, these drugs have been shown to slow the decline in lung function in patients with IPF but do not improve the survival rate of patients. Therefore, it is necessary to further identify the prognostic markers and therapeutic targets of IPF.

Epithelial mesenchymal transition (EMT) is a biological process in which epithelial cells lose adhesion and apical–basal polarity, change their shape, undergo great cytoskeletal changes, and acquire some interstitial features, such as invasion, migration, and production of the extracellular matrix (Acloque et al., 2009; Jolly et al., 2018). Although the detailed pathogenesis of IPF is not completely clear, increasing evidence has revealed that there is a close relationship between EMT and IPF (Chilosi et al., 2017; Yao et al., 2019). For example, it has been found that the degradation products of the extracellular matrix (matrikines) play a signaling role in addition to their structure and function, and these signaling molecules have been shown to play a central role in the fibrotic response of IPF by regulating epithelial cell repair, promoting fibroblast recruitment and myofibroblast activation (Hewlett et al., 2018). Stromal cells in IPF are often found to express both epithelial and stromal markers (Gjorevski et al., 2012). In addition, there is increasing evidence that some fibroblast growth factors are significantly upregulated in IPF (Coffey et al., 2013; MacKenzie et al., 2015). Similarly, calmodulin-positive pleural mesothelial cells (PMCs) were found to exit in the removed lung tissues of IPF patients, and the number of calmodulin-positive PMCs correlated with the degree of fibrosis, suggesting that trafficking in PMCs may play a pathogenic role in IPF through regulating mesothelial–interstitial transformation and invasion of the lungs (Mubarak et al., 2012; Batra and Antony, 2015). More importantly, dysregulation of the activation of lung repair processes by mechanical stress may lead to lung remodeling of IPF through EMT (Morbini et al., 2011).

It has been found that the injury of lung epithelial cells and the extracellular matrix leads to the release of cytokines, chemokines, and growth factors (Selman et al., 2001), indicating that EMT may also be related to IPF immune response. Gradually growing evidence suggests that immune response plays an important role in IPF. For instance, there is a significant increase in levels of pro-inflammatory cytokines such as TNF-α and IL-8 in the lungs of patients with IPF (Kapanci et al., 1995), and TNF-α has been shown to be important in bleomycin-induced fibrosis (Ortiz et al., 1998). On the other hand, in fact, the importance of immune response in IPF has been shown by genetic studies, and the polymorphism of immune-related genes, such as genes encoding Toll-like receptor 3 (TLR3), Toll-interacting protein (TOLLIP), and interleukin-1 receptor antagonist (IL-1RA) proteins, are closely related to the risk or severity of IPF (Whyte et al., 2000; O’Dwyer et al., 2013). Moreover, bronchoalveolar lavage fluid cells obtained from IPF patients during the onset of the disease produced higher levels of IL-1RA, CCL18, and other M2-related chemokines than cells obtained from the same patients outside the onset of the disease, and the higher the content of CCL18, the higher the seizure (Schupp et al., 1932). Furthermore, some studies have found that the increase in IL-18 participates in bleomycin-induced PF development by regulating EMT in a Snail-1-dependent manner (Zhang et al., 2019). Therefore, further research is needed to better clarify the mechanism of EMT in IPF and the correlation between EMT and immunity, so as to provide a theoretical basis for the future development of diagnosis and prognosis biomarkers or treatment.

In this study, we first found that EMT-related genes are mainly involved in immune-related biological processes and pathways, and further identified EMT- and immune-related genes. Moreover, through univariate Cox and least absolute shrinkage and selection operator (LASSO) regression analyses, we established the EMT- and immune-related gene signatures to predict the prognosis of patients with IPF. Therefore, this study may contribute to a better understanding of the relationship between EMT and immunity and contributes to the treatment of IPF.

## Methods

### Acquisition and Correction of Datasets

Two microarray expression profiling datasets (GSE70866 and GSE28221) were acquired from the Gene Expression Omnibus (GEO) database (http://www.ncbi.nlm.nih.gov/geo/). The dataset of GSE70866 contains gene expression data of 176 bronchoalveolar lavage cells from IPF patients with survival information, including gene expression data of 112 IPF patients obtained by the GPL14550 platform (Agilent-028004 SurePrint G3 Human GE 8 × 60K Microarray, Agilent Technologies) and gene expression data of 64 IPF patients acquired by the GPL17077 platform (Agilent-039494 SurePrint G3 Human GE v2 8 × 60K Microarray). The GSE28221 dataset includes gene expression data of 119 peripheral blood mononuclear cells of from IPF patients with survival information, including gene expression data of 74 IPF patients obtained by the GPL5175 platform (Affymetrix Human Exon 1.0 ST Array, Affymetrix), and gene expression data of 45 IPF patients acquired by the GPL6480 platform (Agilent-014850 Whole Human Genome Microarray 4 × 44K G4112F). Surrogate variable analysis (SVA) 3.14 package in R was selected to eliminate the batch effect in each dataset by using default parameters (Parker et al., 2014). After batch elimination, the GSE70866 and GSE28221 datasets were defined as the training set and the validation set, respectively. Moreover, the 200 epithelial–mesenchymal transition (EMT)-related genes (Supplementary Material S1) in the “HALLMARK_EPITHELIAL_MESENCHYMAL_TRANSITION” were downloaded from the Molecular Signatures Database (MSigDB version 6.0).

### Identification of Epithelial-Mesenchymal Transition-Related Genes

First, gene set variation analysis (GSVA) was performed using GSVA 1.36.3R 4.1.2 package by using default parameters to obtain EMT scores of all IPF patients in the training set (Hänzelmann et al., 2013). Next, patients were divided into the high-EMT score and low-EMT score groups based on the optimal cutting point of the EMT score acquired by survminer R package. Unified Manifold Approximation and Projection (UMAP), a non-linear dimensionality reduction algorithm that can divide or condense a group of patients into a series of distinct clusters, according to the given hallmarks or signatures, was applied to observe the distribution of IPF patients based on the expression levels of 200 EMT-related genes. Moreover, Kaplan–Meier (K–M) survival analysis was used to further investigate the correlation between the EMT score and the prognosis of IPF patients, and the log rank test was used to perform statistical analysis (*p* val < 0.05). Finally, differentially expressed genes (DEGs) between the high-EMT score and low-EMT score groups were identified using limma 3.44.3 package in R (doi: 10.1093/nar/gkv007) and were defined as EMT-related genes. The screening criterion for DEGs was |log2 (fold change)| > 1 and adj. *p* val < 0.05. The volcano plot and heatmap of the DEGs were visualized using ggplot2 3.3.2 package (Hänzelmann et al., 2013) and pheatmap 0.7.7 package (Hänzelmann et al., 2013) in R.

### Identification of Epithelial-Mesenchymal Transition- and Immune-Related Genes

Immune scores of all IPF patients in the training set were calculated using the Estimation of STromal and Immune cells in MAlignant Tumors using Expression data (ESTIMATE) algorithm 1.0.13 (Yoshihara et al., 2013). According to the optimal cutting point of the immune score acquired using survminer 0.4.9R package by setting minprop = 0.3 (Li et al., 2021a), patients were divided into the high-immune score and low-immune score groups. Next, K–M survival analysis was used to investigate the correlation between the immune score and the prognosis of IPF patients, and the log rank test was used to perform statistical analysis (*p* val < 0.05). DEGs between the high-immune score and low-immune score groups were identified using limma package in R and were defined as immune-related genes. The screening criterion for DEGs was |log2 (fold change)| > 1 and adj. *p*. val < 0.05. The volcano plot and heatmap of the DEGs were visualized using ggplot2 package (Yoshihara et al., 2013) and pheatmap package (Yoshihara et al., 2013). Finally, EMT- and immune-related genes were identified using Venn diagram by overlapping EMT- and immune-related genes.

### Functional, Pathway, and Disease Ontology Enrichment Analysis

clusterProfiler 3.14 package in R was selected to perform Gene Ontology (GO), including biological processes (BP), molecular function (MF), cellular component (CC), functional annotation, disease ontology (DO), and Kyoto Encyclopedia of Genes and Genomes (KEGG) pathway enrichment analysis (Yu et al., 2012). *p* val < 0.05 and Q val < 0.05 were regarded as significant.

### Construction and Validation of a Epithelial-Mesenchymal Transition- and the Immune-Related Gene Signature

First, prognosis-associated genes were screened from EMT- and immune-related genes in the training set (*p* val < 0.05) using univariate Cox regression analysis by using survival 3.2–3R package. Next, least absolute shrinkage and selection operator (LASSO) regression analysis was selected to remove false positive genes in the training set by using glmnet 4.1–3R package (Friedman et al., 2010). Moreover, multivariate Cox regression was used to construct an optimal EMT- and immune-related gene signature in the training set. Thus, the risk score of each IPF patient in the training set and validation set was calculated based on gene expression and corresponding coefficient acquired from the multivariate Cox regression model, separately. According to risk scores, IPF patients in the training set and validation set were classified into low-risk and high-risk groups based on optimal cutting point of the risk score in the training and validation set, separately. Furthermore, K–M and time-dependent receiver performance (ROC) curves were used to assess the validity and applicability of the EMT- and immune-related gene signatures applying survival (Yoshihara et al., 2013) and survivalROC 1.0.3 (Heagerty and Zheng, 2005) R package.

To better predict the prognosis of IPF patients, we established a nomogram for predicting 1-, 2- and 3-year survival of IPF patients using in the training set. First, based on the previously constructed gene signature, the prediction days were set to 365, 730, and 1,095 days. Next, the nomogram function was selected to construct the nomogram by setting lp = T. Next, the calibration curve was selected to access the predictive efficiency of the nomogram.

### Gene Set Enrichment Analysis

Gene set enrichment analysis (GSEA), a method for assessing whether a set of genes exhibit a statistically significant, and concordant difference between two different biological conditions (Damian and Gorfine, 2004) was performed based on the genes expression matrix of all genes between low-risk and high-risk groups in the training set by GSEA V4.1.0 using default parameters, and pathways in “c2.cp.kegg.v7.2.symbols.gmt” were used as the reference gene set. *p* val was considered significant enrichment.

### Immune Cell Infiltration Related to the Epithelial-Mesenchymal Transition- and Immune-Related Gene Signature

Cell-type Identification By Estimating Relative Subsets Of RNA Transcripts (CIBERSORT) was used to analyze the immune cell composition of all patients in the low-risk and high-risk groups based on a validated leukocyte gene signature matrix, which includes 547 genes and 22 human immune cell subpopulations in the training set (Newman et al., 2015). Moreover, the Wilcoxon test was applied to test whether there were significant differences of 22 immune cells between low-risk and high-risk groups, and *p* val < 0.05 was regarded as a statistical difference. Moreover, we also investigated the correlation between immune cells and genes in the EMT- and immune-related gene signatures based on Pearson correlation analysis, and *p* val < 0.05 was considered as statistical difference.

### Patient and Tissue Preparation

We selected the blood of 10 IPF patients and 10 healthy people to perform quantitative PCR detection on the genes screened in this study. Blood samples were donated anonymously from patients with confirmed IPF in the Third Provincial Hospital of Shandong Province and Jining People’s Hospital. The experimental validation data were obtained from the sample database of the Pediatric Research Institute of Tianjin Children’s Hospital, and there were no samples directly from patients, so the approval of the Institutional Ethics Committee of the hospital was not required. All experimental operations were carried out in accordance with relevant guidelines and regulations and were repeatedly verified by professional laboratory researchers.

### RNA Isolation and Quantitative Real-Time Polymerase Chain Reaction

Quantitative real-time polymerase chain reaction (qRT-PCR) was performed to detect IL1R2, S100A12, and CCL8 expression using American Bio-Rad Bole T100 gradient PCR instrument. After total RNA extraction with TRIzol reagent (TaKaRa), cDNA reverse transcription and qRT-PCR were performed. The primer sequence and annealing temperature are summarized in Supplementary Table S1.

## Results

### Identification of Epithelial-Mesenchymal Transition-Related Genes

**FIGURE 1:**
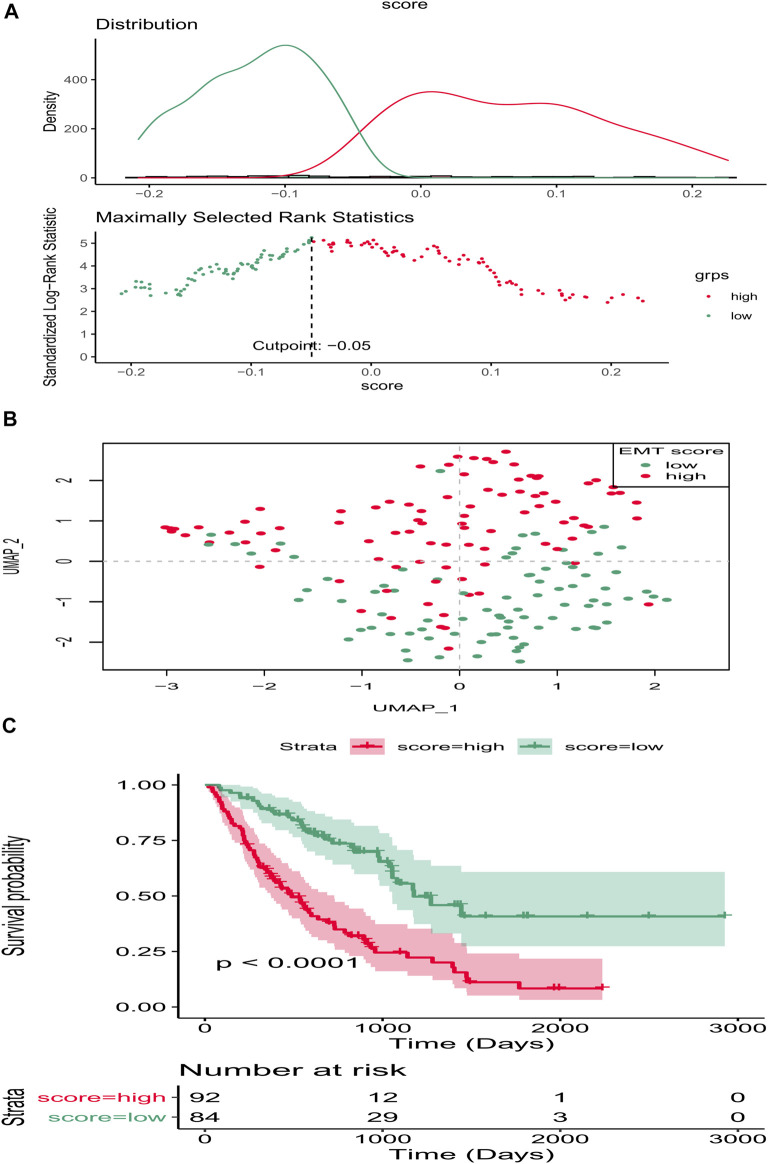
EMT score affects the prognosis of IPF in the training set. **(A)** Optimal cutting point analysis suggested that the optimal cutting point of the EMT score was equal to −0.05 in the training set. Green represents low score of EMT, and red represents high score of EMT. **(B)** UMAP cluster of IPF patients in the training set based on EMT-related genes. **(C)** Survival curve of high- and low-EMT score groups.

**FIGURE 2:**
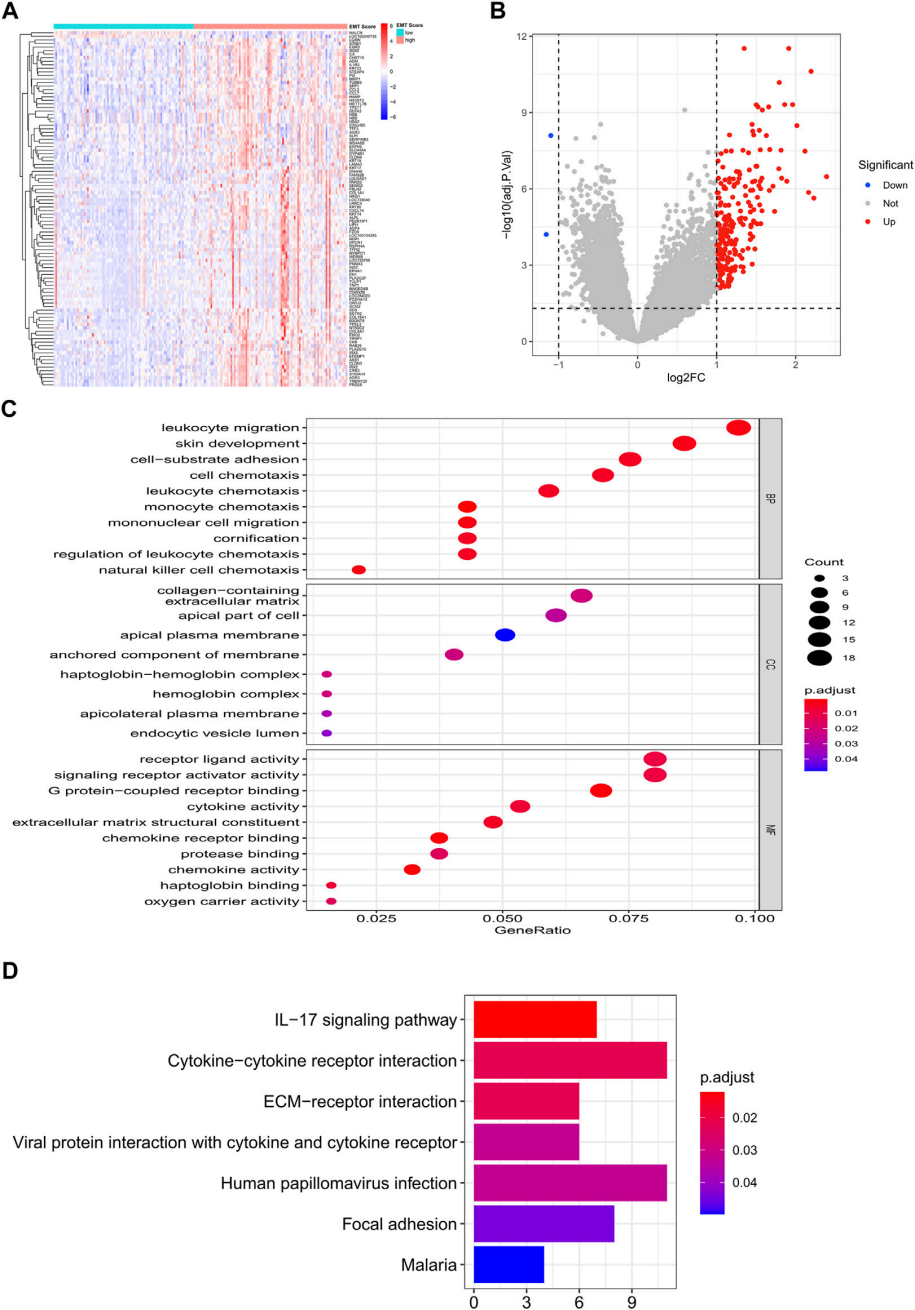
Identification of EMT-related genes in the training set. **(A)** Heatmap of DEGs between patients of the EMT score-high group and patients in the immune score-low group. **(B)** Volcano plot of DEGs between patients of the EMT score-high group compared with patients in the immune score-low group. **(C)** GO enrichment analysis of EMT-related genes. **(D)** KEGG enrichment analysis of EMT-related genes.

Clearly, PCA cluster showed that all samples in the training and validation sets were available for subsequent analyses after batch effect treatment (Supplementary Figure S1). The optimal cutting point analysis suggested that the optimal cutting point of the EMT score was equal to -0.05 in the training set (Figure 1A). Thus, according to the optimal cutting point, 176 IPF patients in the training set were divided into EMT score-high and EMT score-low groups, including 92 and 84 IPF patients in the EMT score-high and EMT score-low groups, separately. UMAP cluster also revealed that 176 patients could be divided into two clusters, and each patient was assigned to the nearest EMT score-high and EMT score-low groups (Figure 1B). Interestingly, the difference of survival between these two groups was statistically significant (*p* < 0.0001), and the prognosis of patients in the EMT score-high group was poorer than those in the EMT score-low group (Figure 1C). Next, a total of 230 DEGs (Supplementary Material S2), including 228 upregulated genes and two downregulated genes in patients of the EMT score-high group compared with patients in the immune score-low group, were identified and defined as EMT-related genes (Figures 2A,B). In addition, functional enrichment analysis revealed that these DEGs were mainly involved in immune-related BPs and signaling pathways, such as monocyte chemotaxis, leukocyte migration, IL-17 signaling pathway, and cytokine–cytokine receptor interaction signaling pathway (Figures 2C,D).

### Identification of Epithelial-Mesenchymal Transition- and Immune-Related Genes

**FIGURE 3:**
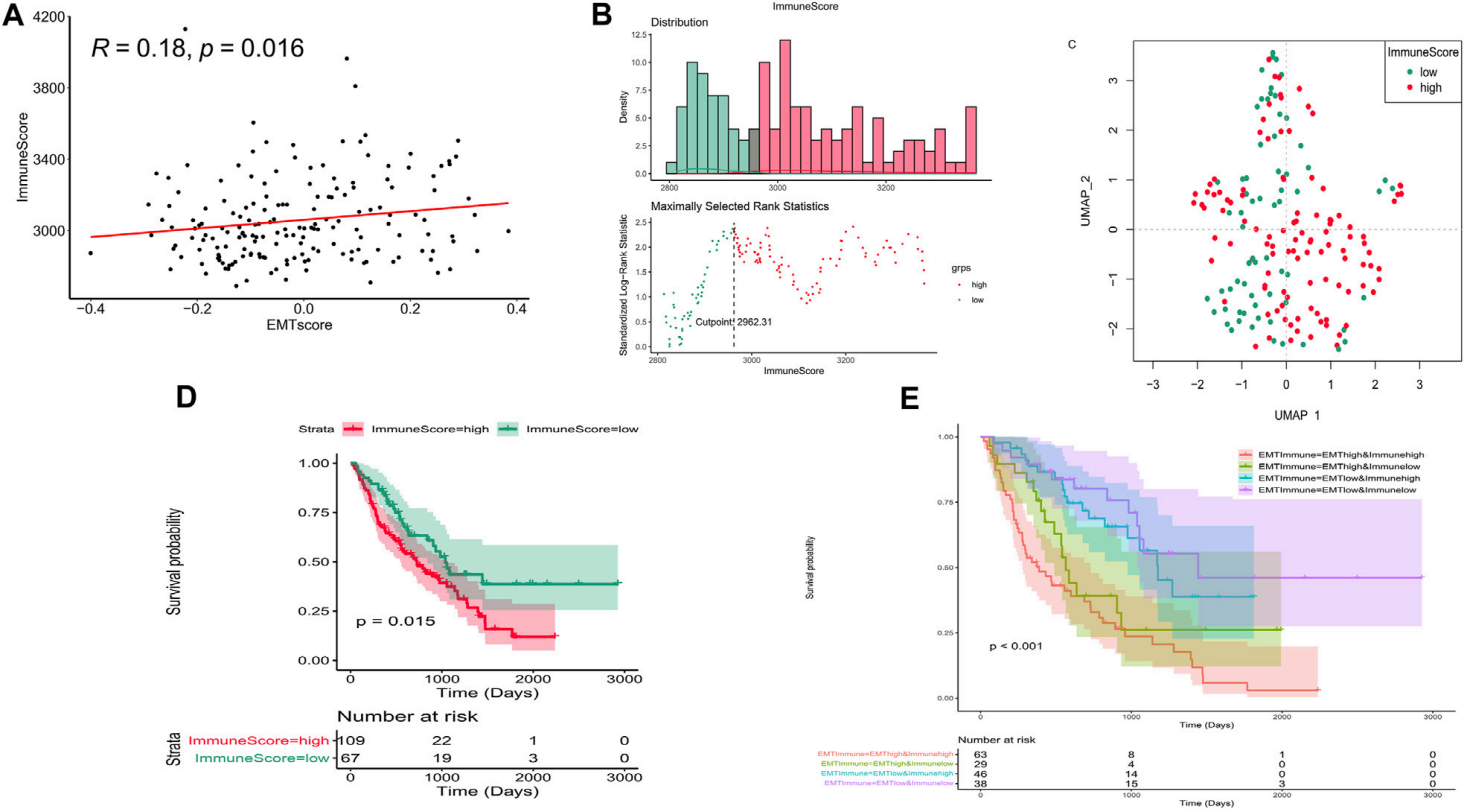
Immune score affect the prognosis of IPF in the training set. **(A)** Correlation between the EMT and immune scores. **(B)** Optimal cutting point analysis suggested that the optimal cutting point of the immune score was equal to 2,962.31 in the training set. Green represents low score of immune, and red represents high score of immune. **(C)** UMAP cluster of IPF patients in the training set based on immune-related genes. **(D)** Survival curve of high- and low-immune score groups. **(E)** Survival curve of high- and low-EMT and immune score groups.

**FIGURE 4:**
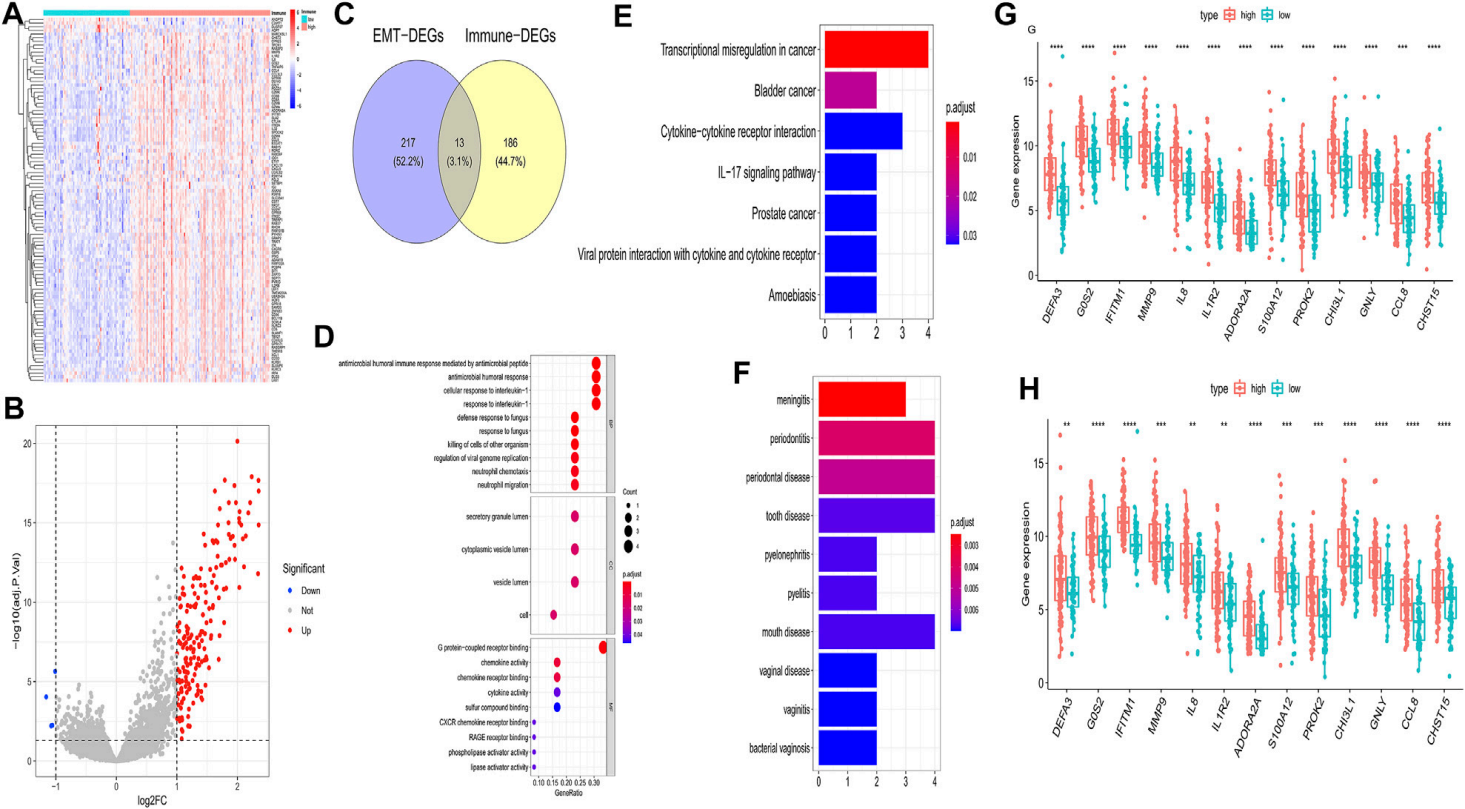
Identification of EMT- and immune-related genes and functional enrichment analysis in the training set. **(A)** Heatmap of DEGs between patients of the immune score-high group and patients of the immune score-low group. **(B)** Volcano plot of DEGs between patients of the immune score-high group and patients of the immune score-low group. **(C)** Venn diagram to identify EMT- and immune-related genes **(D)** GO enrichment analysis of EMT- and immune-related genes. **(E)** KEGG enrichment analysis of EMT- and immune-related genes. **(F)** DO enrichment analysis of EMT- and immune-related genes. **(G)** Differences of 13 genes in G-GSE70866 between high- and low-EMT score groups. **(H)** Differences of 13 genes in H-GSE70866 between high- and low-immune score groups.

To further investigate the influence on immune of these EMT-related genes in IPF patients and screen EMT and immune-related genes, we calculated the immune scores of all IPF patients in the training set using the ESTIMATE algorithm. We examined the correlation between the EMT and immune scores and found that they were positively correlated (Figure 3A). The optimal cutting point analysis suggested that the optimal cutting point of the immune score was equal to 2,962.31 in the training set (Figure 3B). Thus, according to the optimal cutting point, 176 IPF patients in the training set were divided into the immune score-high and immune score-low groups, including 109 and 67 IPF patients in the immune score-high group and immune score-low group, separately. UMAP cluster also showed that 176 patients could be divided into two clusters, and each patient was assigned to the nearest immune score-high and EMT score-low groups (Figure 3C). Pleasingly, the difference of survival between these two groups was markedly significant (*p* = 0.015), and the prognosis of patients in the immune score-high group was worse than that in the immune score-low group (Figure 3D). Notably, we also found that patients with the high EMT and immune scores had the lowest prognosis, while patients with low EMT and immune scores had the best prognosis (Figure 3E). Subsequently, a total of 199 DEGs (Supplementary Material S3), including 195 upregulated genes and four downregulated genes in patients of the immune score-high group compared with patients in the immune score-low group, were identified and defined as immune-related genes (Figures 4A,B). After overlapping EMT-and immune-related genes, 13 EMT- and immune-related genes were identified (Figure 4C, Supplementary Table S2). Finally, GO annotation suggested that these 13 EMT- and immune-related genes were mainly involved in antimicrobial humoral response, cellular response to interleukin-1, neutrophil chemotaxis-related BPs, secretory granule lumen, and cytoplasmic vesicle lumen-related CCs, and G protein-coupled receptor binding and chemokine activity (Figure 4D). On the other hand, KEGG enrichment analysis showed that these 13 EMT- and immune-related genes were mainly associated with cytokine–cytokine receptor interaction and IL-17 signaling pathway (Figure 4E). DO enrichment analysis suggested that these 13 EMT- and immune-related genes were mainly correlated with inflammation-related diseases, such as meningitis, pyelitis, and periodontitis (Figure 4F). Interestingly, we found that these 13 genes were upregulated both in the high EMT and immune groups (Figures 4G,H). Thus, these 13 EMT- and immune-related genes might affect IPF by regulating immune response.

### Construction and Validation of Epithelial-Mesenchymal Transition- and Immune-Related Gene Signature

**FIGURE 5:**
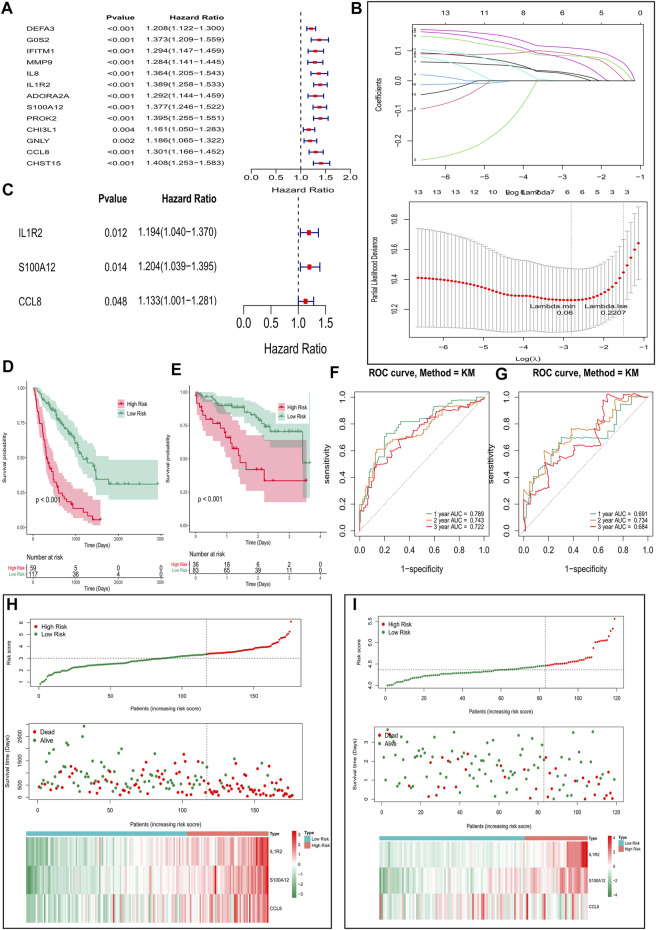
Construction and validation of the EMT- and immune-related gene signatures based on EMT- and immune-related genes. **(A)** Univariate Cox regression analysis identified genes related to the prognosis of IPF patients in the training set. **(B)** Screening of characteristic genes by LASSO regression analysis. **(C)** Multivariate Cox regression analysis to construct the EMT- and immune-related gene signatures in the training set. **(D,E)** Survival curve of high- and low-risk groups in the training set **(D)** and validation set **(E)**. **(F,G)** ROC curves evaluated the efficiency of the risk signature for predicting 1-, 2-, and 3-year survival in the training set **(F)** and the validation set **(G)**. **(H,I)** Three genes expression profiles, the risk score distribution, and patients’ survival status in the training set **(H)** and the validation set **(I)**.

**FIGURE 6:**
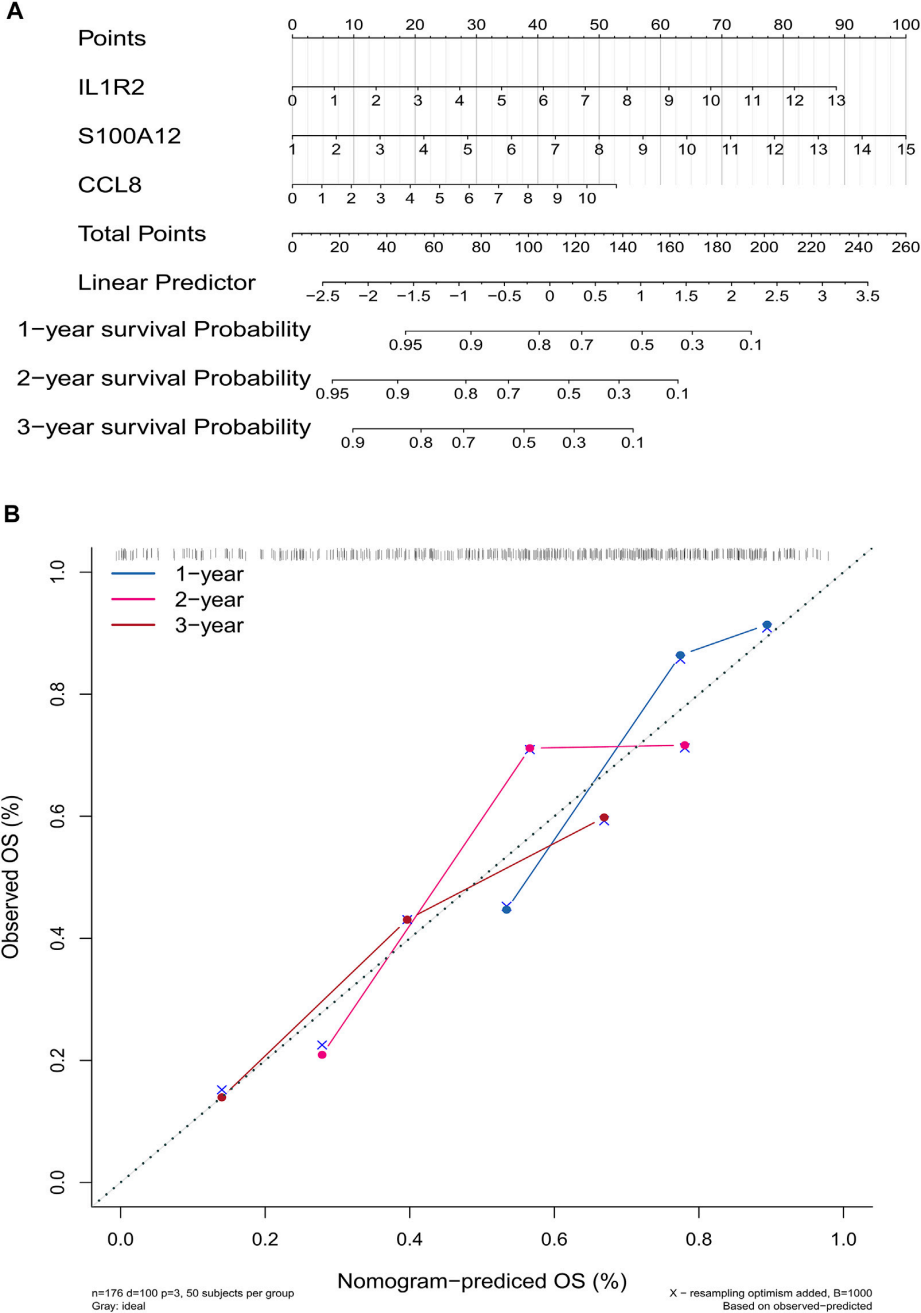
Construction and evaluation of a nomogram for predicting 1-, 2-, and 3-year survival rates of IPF patients in the training set. **(A)** Nomogram for predicting 1-, 2-, and 3-year survival of IPF patients. **(B)** Calibration curves showing the probability of 1-, 2-, and 3-year survival between the prediction and the observation.

To establish a gene signature for predicting the prognosis of IPF patients, we first performed univariate Cox regression analysis based on expression and survival data of 13 EMT- and immune-related genes in the training set. Interestingly, we found that 13 EMT- and immune-related genes were related to the prognosis of IPF patients (Figure 5A). Moreover, six EMT- and immune-related genes, including DEFA3, IL1R2, ADORA2A, S100A12, PROK2, and CCL8, were screened at a lambda.min of 0.06 (Figure 5B). Finally, the EMT- and immune-related gene signatures were established based on IL1R2, S100A12, and CCL8 expression and their corresponding coefficient obtained from multivariate Cox regression analysis (Figure 5C and Table 1). The risk score of each IPF patients in the training and validation sets was calculated using the following formula: risk score = (0.012) × IL1R2 expression + 0.014 × S100A12 expression + 0.048 × CCL8 expression. Subsequently, patients in the training set were divided into high-risk and low-risk groups according to the optimal cutoff value (3.342592) of risk score. As shown in Figure 5D, the K–M survival curve showed a significant difference of survival for IPF patients between the high and low-risk groups (*p* val < 0.001), and patients in the high-risk group had a lower survival rate in the training set. The area under ROC curves for predicting 1-, 2-, and 3-year survival were greater than 0.7 in the training set (Figure 5F). Notably, IL1R2, S100A12, and CCL8 presented higher expression in patients of the high-risk group than patients in the low-risk group in the training set (Figure 5H). Furthermore, patients in the low-risk group also showed a better prognosis than patients in the high-risk group in the validation set (Figure 5E). The area under ROC curves for predicting 1-, 2-, and 3-year survival were also greater than 0.6 validation set (Figure 5G), and IL1R2, S100A12, and CCL8 also presented higher expression in patients of the high-risk group than patients in the low-risk group (Figure 5I). Therefore, the EMT- and immune-related gene signatures were a good predictor of prognosis for IPF patients. Finally, to predict the prognosis of patients more intuitively, we constructed a nomogram for predicting 1-, 2- and 3-year survival of IPF patients in the training set based on IL1R2, S100A12, and CCL8 (Figure 6A), and the calibration curves showed that the observed and true values coincide well (Figure 6B). Thus, the nomogram based on IL1R2, S100A12, and CCL8 presented a good accuracy for predicting 1-, 2- and 3-year survival of IPF patients.

**TABLE 1:** Results of multivariate Cox regression analysis.

id	Coef	HR	HR.95L	HR.95H	*p* value
IL1R2	0.177105131	1.193756587	1.039919913	1.370350517	0.011867388
S100A12	0.18550844	1.20383036	1.039012538	1.394793116	0.01353368
CCL8	0.12467842	1.132784113	1.001368767	1.281445847	0.047512265

### GSEA of the Epithelial-Mesenchymal Transition- and Immune-Related Gene Signature

**FIGURE 7:**
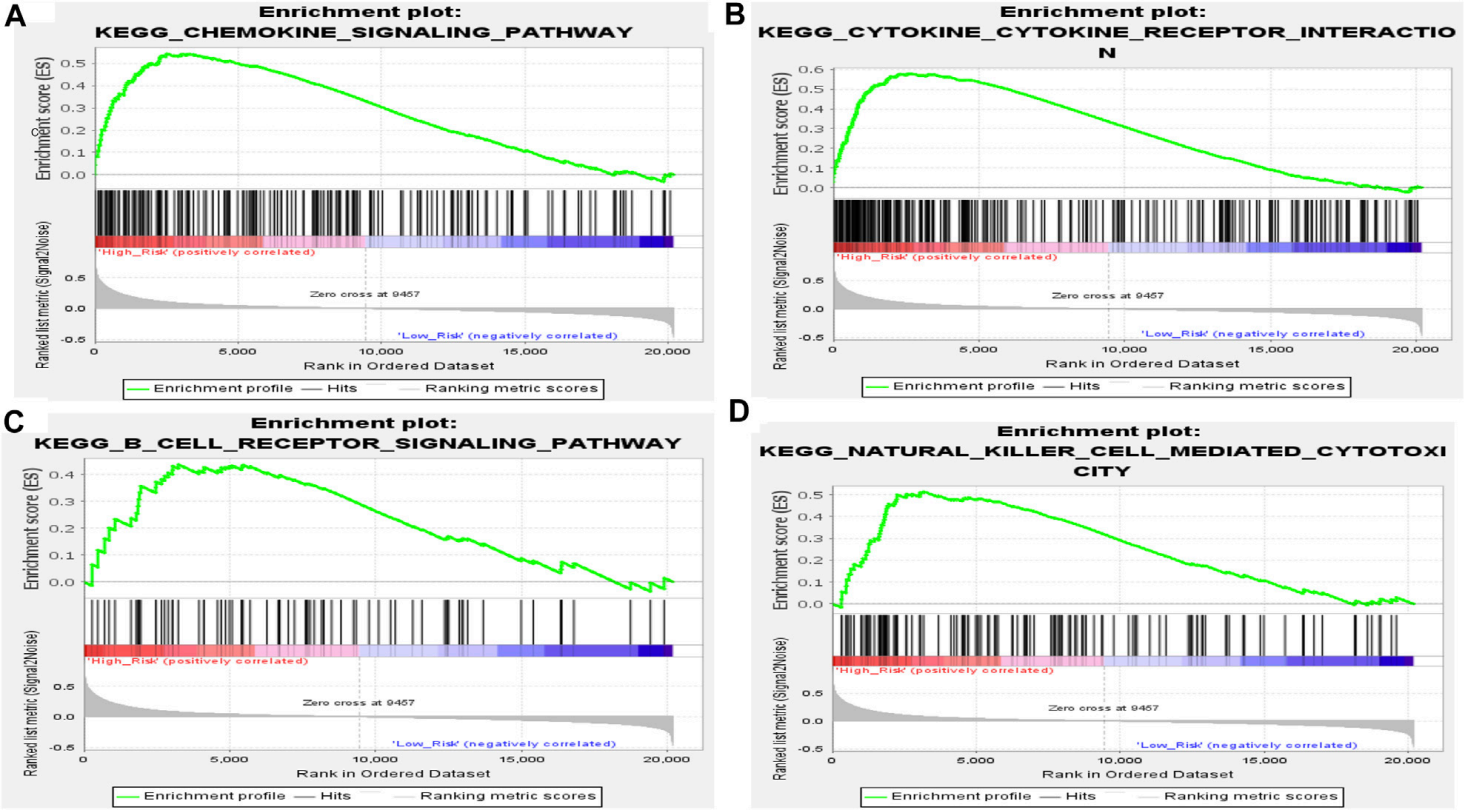
Results of GSEA of KEGG pathway in high- and low-risk groups in the training set. **(A)** Chemokine signaling pathway. **(B)** Cytokine–cytokine receptor interaction. **(C)** B-cell receptor signaling pathway. **(D)** Natural killer cell-mediated cytotoxicity.

To further investigate the function of the EMT- and immune-related gene signatures, GSEA was performed based on the expression matrix of all genes between the high-risk and the low-risk groups. As expected, we found that immune-related pathways, including chemokine signaling pathway, cytokine–cytokine receptor interaction, B-cell receptor signaling pathway, and natural killer cell-mediated cytotoxicity, were activated in the high-risk group of patients (Figure 7). Therefore, we speculated that IL1R2, S100A12, and CCL8 may play key roles in IPF by affecting immune response.

### Immune Cell Infiltration Related to the Epithelial-Mesenchymal Transition- and Immune-Related Gene Signature

**FIGURE 8:**
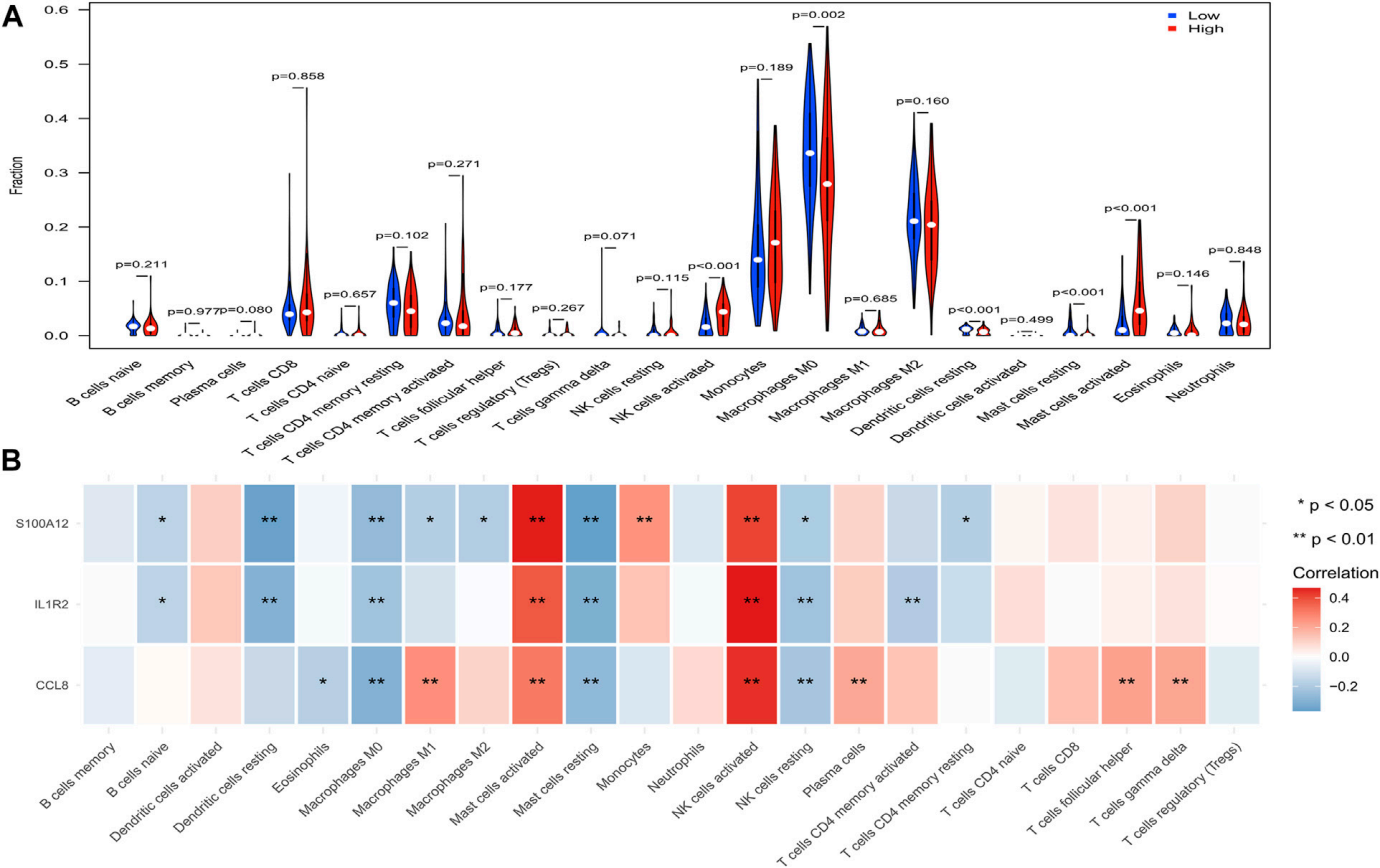
Analysis of immune infiltrating cells in high- and low-risk groups in the training set. **(A)** Comparison of infiltrated immune cells in high- and low- risk groups. **(B)** Correlation between IL1R2, S100A12, and CCL8 and infiltrated immune cells.

To further explore the effect of the EMT- and immune-related gene signatures on immunity, CIBERSORT was used to investigate the immune cell infiltration between patients in the high-risk and low-risk groups. Just as expected, we found that NK cells activated, macrophages M0, dendritic cells resting, mast cells resting, and mast cells activated were significantly different between patients in the high-risk and low-risk groups (Figure 8A). Clearly, NK cells activated and mast cells activated were infiltrated more in the high-risk group, while macrophages M0, dendritic cells resting, and mast cells resting infiltrated more in the low-risk group (Figure 8A). In addition, IL1R2, S100A12, and CCL8 were positively associated with NK cells activated and mast cells activated but negatively associated with macrophages M0, dendritic cells resting, and mast cells resting (Figure 8B). Thus, IL1R2, S100A12, and CCL8 might affect IPF by regulating NK cells activated, macrophages M0, dendritic cells resting, mast cells resting, and mast cells activated.

### The mRNA Expression of IL1R2, S100A12, and CCL8

**FIGURE 9:**
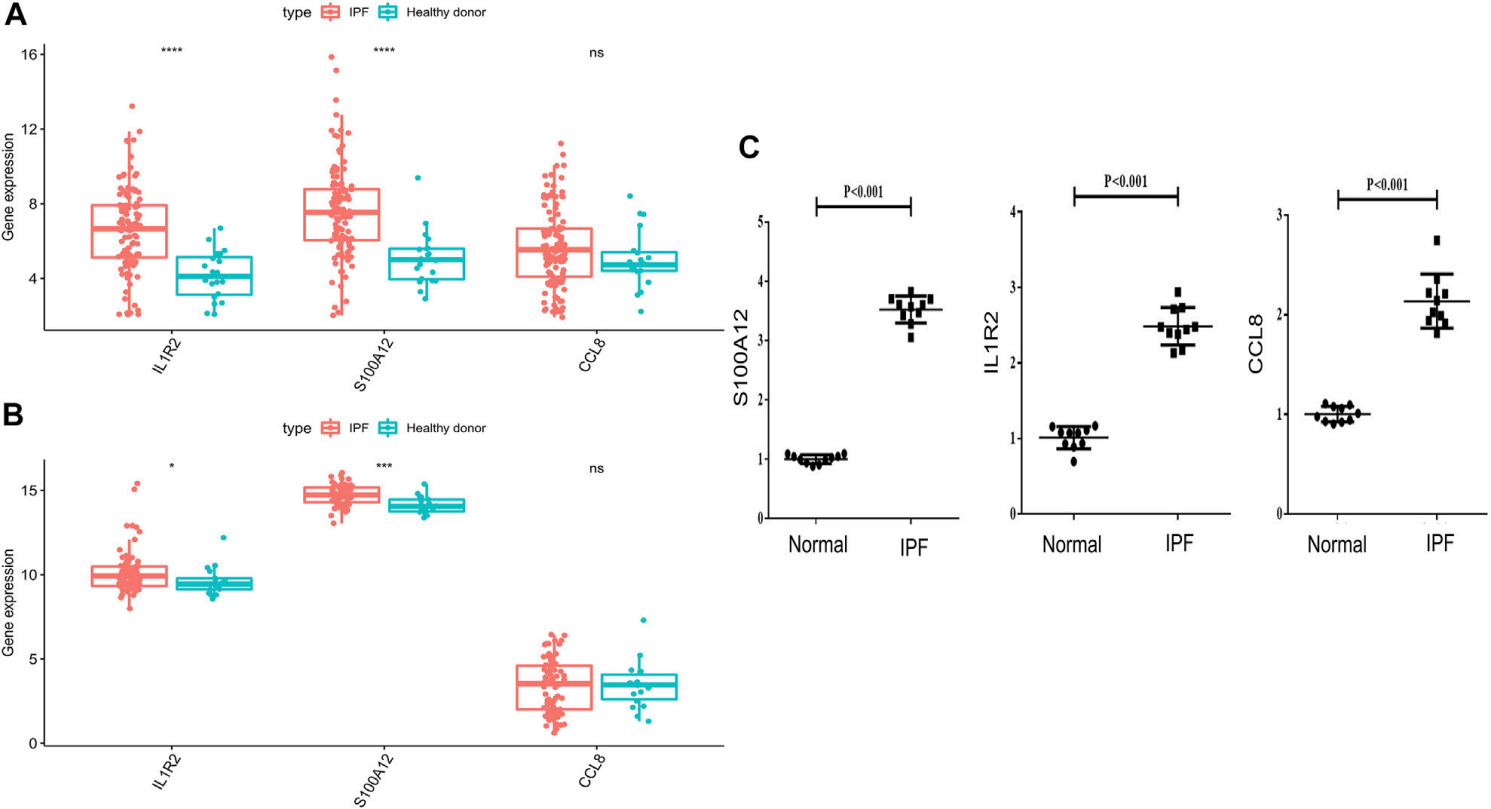
Validation of mRNA expression levels of IL1R2, S100A12, and CCL8 with the GSE708066 and GSE28221 datasets and qRT-PCR. **(A)** Differences of three genes in GSE70867-GPL14550. **(B)** Differences of three genes in GSE28221-GPL6480. **(C)** mRNA expression levels of IL1R2, S100A12, and CCL8 tested by qRT-PCR.

To explore the expression of IL1R2, S100A12, and CCL8 in IPF, we first compared the expression of IL1R2, S100A12, and CCL8 between normal samples and IPF patients in GSE708066 and GSE28221 datasets. Interestingly, we found that IL1R2 and S100A12 significantly upregulated in peripheral blood mononuclear cells and bronchoalveolar lavage cells (Figures 9A,B). Although there was no significant difference for CCL8, it was more highly expressed in IPF (Figures 9A,B). Moreover, we collected the whole blood of 10 IPF patients and 10 healthy individuals and analyzed the mRNA expression levels of IL1R2, S100A12, and CCL8 by qRT-PCR. The results suggested that the expression levels of IL1R2, S100A12, and CCL8 in IPF patients were higher than those in healthy people (Figure 9C). Therefore, IL1R2, S100A12, and CCL8 might play key roles in IPF.

## Discussion

IPF is the most common subtype of interstitial lung disease in the world (1). However, among the various interstitial lung disease subtypes, its prognosis is the worst, with the median survival time after diagnosis is 3–5 years (Raghu et al., 2006; Fernández Pérez et al., 2010; Martinez et al., 2017). The treatment of IPF has been hindered by the lack of understanding of the pathophysiology and true natural history of the disease. Currently, there are only two FDA-approved drugs for IPF (pirfenidone and nindanib), which do not prevent or reverse the progression of the disease or reduce mortality; they can only moderately reduce the rate of deterioration of lung function (King et al., 2014; Richeldi et al., 2014). In addition, IPF is a highly heterogeneous disease with a great change in its natural history (Kim et al., 2006; Kim et al., 2015). Thus, the course of individual patients is difficult to predict (Ley et al., 2011; Mura et al., 2012). For example, some patients with IPF deteriorate rapidly, while others develop much more slowly (Ley et al., 2011; Raghu et al., 2011). For a long time, due to the lack of effective prognostic indicators, it is difficult to accurately track and evaluate the prognosis of IPF, which to a certain extent led to the poor prognosis of IPF. Therefore, the development of suitable prognostic markers is an urgent need for clinical treatment of IPF.

In this study, we first found that the EMT and immune scores affect the prognosis of patients with IPF. Moreover, we identified 230 EMT- and 199 immune-related genes. Notably, we found that most EMT-related genes were upregulated in the high-EMT group, and most immune-related genes were upregulated in the high-immune group, indicating that upregulated EMT- and immune-related genes may be positively associated with EMT and immunity, separately. Furthermore, 13 EMT- and immune-related genes were screened by overlapping EMT- and immune-related genes. Thus, we speculated that these 13 genes may be linked to both immune and EMT in IPF. Finally, through univariate Cox and LASSO regression analyses, we constructed an EMT- and immune-related gene signatures based on IL1R2, S100A12, and CCL8 by using their expression in bronchoalveolar lavage cells of IPF patients, and K–M and ROC analyses revealed that the EMT- and immune-related gene signatures could well predict the 1-, 2-, and 3-year survival of IPF patients. Similarly, we found that this signature also can predict patient prognosis based on peripheral blood data, indicating its wide applicability. Therefore, the signature based on IL1R2, S100A12, and CCL8 may help clinical prediction of prognosis for IPF patients by detecting the expression of these three genes in peripheral blood. Comparing with previous signatures (Li et al., 2021b; Qiu et al., 2021), we developed a nomogram that directly predicts patient prognosis based on the expression of IL1R2, S100A12, and CCL8.

Interleukin-1 receptor type 2 (IL1R2), through competitive binding with IL1 β, prevents it from binding to IL1R1, thus blocking the signal transduction of IL1 β in inflammatory diseases and acting as a bait receptor (Molgora et al., 2018). In aseptic inflammation caused by necrosis, IL1R2 can also be used as an inhibitor of intracellular pro-IL1 α (Zheng et al., 2013). Consistent with our results, IL1R2 was found to be upregulated in IIP lung tissues compared with normal lung tissues, and in different IIP subtypes (Chen et al., 2014). In addition, ILIR2 can impair the ability of endometrial epithelial cells to counteract the local effects of IL-1 (Herrmann-Lavoie et al., 2007). IL1R2 expressed by epithelial cells acts as a homeostatic regulator in the remission of ulcerative colitis (Mora-Buch et al., 2016). Therefore, we speculated that IL1R2 may play an important role in IPF by regulating immune response.

It has been found that S100A12 expression in lungs, bronchoalveolar lavage fluid, and serum of patients with acute respiratory distress syndrome (ARDS) is higher than that in healthy controls (Lorenz et al., 2008; Kikkawa et al., 2010). S100A12 is widely expressed in the local inflammatory site of cystic fibrosis. It is a serum marker of acute exacerbation of infection. The local expression of S100A12 indicates that the protein can promote inflammation during airway inflammation and may be a new target of anti-inflammatory therapy (Foell et al., 2003). S100A12 has the function of pro-inflammatory cytokines, which is related to the fibrotic process of skin scar, and can be used as a potential therapeutic target for skin scar (Zhao et al., 2017). Consistent with our study, S100A12 has been reported to be associated with neutrophil recruitment and activation and is associated with a significant deterioration of IPF, which is upregulated and a prognostic factor (Lu et al., 2021) in patients with IPF. It was also found that S100A12 affected the prognosis of patients with IPF (Lu et al., 2021). The high level of S100A12 protein in serum is related to the poor prognosis of overall survival, non-transplant survival, and progression-free survival in patients with IPF (Richards et al., 2012). Therefore, our study further confirms that S100A12 affects the prognosis of patients.

Chemokine C-C motif ligand 8 (CCL8), also known as monocyte chelating protein (MCP)-2, is expressed by monocytes/macrophages in inflammatory tissues stimulated by T-cell accessory pathways of interferon (IFN) and other pro-inflammatory cytokines, or through innate mechanisms (Ragno et al., 2001; Van Damme et al., 1994) when exposed to fungi, viruses, and bacteria. CCL8 can recruit gamma/delta T cells, which preferentially express IL-17F and synergistically enhance neutrophil chemotaxis (Gouwy et al., 2004) in the presence of IL-8. In addition, Lee et al. (2017) through the analysis of transcriptome data of patients with IPF, it is also found that CCL8 is upregulated in IPF and can be used for the diagnosis and survival prediction of IPF. Therefore, the role of CCL8 in IPF is worthy of further study.

In addition, functional enrichment analysis in high- and low-risk groups showed that immune-related pathways, such as the chemokine signaling pathway, cytokine–cytokine receptor interaction, B-cell receptor signaling pathway, and natural killer cell-mediated cytotoxicity, were significantly activated in high-risk groups, and there were significant differences in infiltration between the high- and low-risk groups (NK cells activated, macrophages M0, dendritic cells resting, mast cells resting, and mast cells activated). In addition, IL1R2, S100A12, and CCL8 were positively correlated with NK cell activation and mast cell activation but negatively correlated with macrophage M0, dendritic cell quiescence, and mast cell quiescence. Studies have shown that alveolar macrophages play a key role in lung balance by removing apoptotic cells and fragments, regulating wound healing, and helping to initiate immune responses (Hussell and Bell, 2014). Pulmonary macrophages have been shown to produce several fibrogenic mediators. A number of studies have shown that pulmonary macrophages produce TGF-β (Khalil et al., 1989; Khalil et al., 1991) in the context of bleomycin-induced fibrosis and in lung sections of patients with IPF. In addition, pulmonary macrophages are not completely suitable for the M1/M2 model, but this model is still helpful to understand the role of macrophages in IPF (Mitsi et al., 2018). Alveolar macrophages from lung transplant donors are relatively homogeneous, but the populations found in IPF patients are highly heterogeneous, and alveolar macrophages from fibrotic lungs express higher levels of fibrosis-related genes (Reyfman et al., 2009). Salonen et al. (1573) found that the mast cells density in the fibrotic area of IPF patients was related to several clinical parameters, and the significant decrease in mast cell count affected the acute deterioration of IPF patients. Shimbori et al. (2019) found that mast cells may be involved in the progression of pulmonary fibrosis. Studies have shown that the proportion and activity of natural killer cells in the lungs of patients with IPF are decreased (Cruz et al., 2021), and cell type analysis based on single-cell transcriptome data also shows that natural killer cells are involved in the progression of IPF (Huang et al., 2021). Dendritic cells can secrete a variety of chemokines and attract different cell types (Piqueras et al., 2006) at different stages of immune response. Therefore, we speculate that IL1R2, S100A12, and CCL8 may affect the development of IPF by regulating these cells.

In conclusion, this study developed and validated the EMT- and immune-related gene signatures based on IL1R2, S100A12, and CCL8. Moreover, we found that IL1R2, S100A12, and CCL8 might regulate IPF pathology by affecting immune response. Furthermore, qRT-PCR suggested that the mRNA expression of IL1R2, S100A12, and CCL8 was upregulated in whole blood of IPF patients compared with normal samples. Therefore, IL1R2, S100A12, and CCL8 may play key roles in IPF. However, this study also has some limitations. Our prognostic model is constructed from public datasets. Although it has been verified by PCR expression test, there are still limited factors such as small sample size, and more prospective studies are needed to verify its predictive ability. In the future, we should continue to pay attention to the role of these three genes in the pathology of IPF through experimental and clinical studies.

## Data Availability

The datasets presented in this study can be found in online repositories. The names of the repository/repositories and accession number(s) can be found at: https://github.com/zhengjiafenglab/IPF.

## Supplementary Material

Supplementary Figure S1 Eliminating the batch effect between different sequencing platforms. **(A,B)** PCA plot before batch effect **(A)** and after batch effect **(B)** in the GSE70866 dataset. **(C,D)** PCA plot before batch effect **(C)** and after batch effect **(D)** in the GSE70866 dataset.

## References

[B1] Acloque H. Adams M. S. Fishwick K. Bronner-Fraser M. Nieto M. A. (2009). Epithelial-mesenchymal Transitions: the Importance of Changing Cell State in Development and Disease. J. Clin. Invest. 119, 1438–1449. (Electronic)). 10.1172/JCI38019 19487820 PMC2689100

[B2] Batra H. Antony V. B. (2015). Pleural Mesothelial Cells in Pleural and Lung Diseases. J. Thorac. Dis. 7, 964. (Print)). 10.3978/j.issn.2072-1439.2015.02.19 26150910 PMC4466423

[B3] Chen H. Fang X. Zhu H. Li S. He J. Gu P. (2014). Gene Expression Profile Analysis for Different Idiopathic Interstitial Pneumonias Subtypes. Exp. Lung Res. 40, 367–379. (Electronic)). 10.3109/01902148.2014.933985 25058599

[B4] Chilosi M. Caliò A. Rossi A. Gilioli E. Pedica F. Montagna L. (2017). Epithelial to Mesenchymal Transition-Related Proteins ZEB1, β-catenin, and β-tubulin-III in Idiopathic Pulmonary Fibrosis. Mod. Pathol. 30, 26–38. (Electronic)). 10.1038/modpathol.2016.147 27586205

[B5] Coffey E. Newman D. R. Sannes P. L. (2013). Expression of Fibroblast Growth Factor 9 in Normal Human Lung and Idiopathic Pulmonary Fibrosis. J. Histochem. Cytochem. 61, 671–679. (Electronic)). 10.1369/0022155413497366 23797050 PMC3753889

[B6] Cruz T. Jia M. Sembrat J. Tabib T. Agostino N. Bruno T. C. (2021). Reduced Proportion and Activity of Natural Killer Cells in the Lung of Patients with Idiopathic Pulmonary Fibrosis. Am. J. Respir. Crit. Care Med. 204, 1535–4970. (Electronic)). 10.1164/rccm.202012-4418LE PMC8491266 34077698

[B7] Damian D. Gorfine M. (2004). Statistical Concerns about the GSEA Procedure. Nat. Genet. 36, 663. (Print)). 10.1038/ng0704-663a 15226741

[B8] Fernández Pérez E. R. Daniels C. E. St. Sauver J. Hartman T. E. Bartholmai B. J. Yi E. S. (2010). Incidence, Prevalence, and Clinical Course of Idiopathic Pulmonary Fibrosis. Chest 137, 129–137. (Electronic)). 10.1378/chest.09-1002 19749005 PMC2803118

[B9] Foell D. Seeliger S. Vogl T. Koch H. G. Maschek H. Harms E. (2003). Expression of S100A12 (EN-RAGE) in Cystic Fibrosis. Thorax 58, 613–617. (Print)). 10.1136/thorax.58.7.613 12832680 PMC1746749

[B10] Friedman J. Hastie T. Tibshirani R. 2010 Regularization Paths for Generalized Linear Models *via* Coordinate Descent. J. Stat. Softw.( 33 1548–7660. (Print). PMC2929880 20808728

[B11] Gjorevski N. Boghaert E. Nelson C. M. (2012). Regulation of Epithelial-Mesenchymal Transition by Transmission of Mechanical Stress through Epithelial Tissues. Cancer Microenvironment 5, 29–38. (Electronic)). 10.1007/s12307-011-0076-5 21748438 PMC3343202

[B12] Gouwy M. Struyf S. Catusse J. Proost P. Van Damme J. (2004). Synergy between Proinflammatory Ligands of G Protein-Coupled Receptors in Neutrophil Activation and Migration. J. Leukoc. Biol. 76, 185–194. (Print)). 10.1189/jlb.1003479 15075362

[B13] Hänzelmann S. Castelo R. Guinney J. (2013). GSVA: Gene Set Variation Analysis for Microarray and RNA-Seq Data. BMC Bioinformatics 14, 7. 10.1186/1471-2105-14-7 23323831 PMC3618321

[B14] Heagerty P. J. Zheng Y. (2005). Survival Model Predictive Accuracy and ROC Curves. Biometrics 61, 92–105. (Print)). 10.1111/j.0006-341X.2005.030814.x 15737082

[B15] Herrmann-Lavoie C. Rao C. V. Akoum A. (2007). Chorionic Gonadotropin Down-Regulates the Expression of the Decoy Inhibitory Interleukin 1 Receptor Type II in Human Endometrial Epithelial Cells. Endocrinology 148, 5377–5384. (Print)). 10.1210/en.2007-0368 17702847

[B16] Hewlett J. C. Kropski J. A. Blackwell T. S. (2018). Idiopathic Pulmonary Fibrosis: Epithelial-Mesenchymal Interactions and Emerging Therapeutic Targets. Matrix Biol. 71-72, 112–127. (Electronic)). 10.1016/j.matbio.2018.03.021 29625182 PMC6146058

[B17] Huang Y. Oldham J. A-O. Ma S. A-O. Unterman A. Liao S. Y. Barros A. J. (2021). Blood Transcriptomics Predicts Progression of Pulmonary Fibrosis and Associated Natural Killer Cells. Am. J. Respir. Crit. Care Med. 204, 1535–4970. (Electronic)). 10.1164/rccm.202008-3093OC PMC8650792 33689671

[B18] Hussell T. Bell T. J. (2014). Alveolar Macrophages: Plasticity in a Tissue-specific Context. Nat. Rev. Immunol. 14, 1474–1741. (Electronic)). 10.1038/nri3600 24445666

[B19] Hutchinson J. Fogarty A. Hubbard R. McKeever T. (2015). Global Incidence and Mortality of Idiopathic Pulmonary Fibrosis: a Systematic Review. Eur. Respir. J. 46, 795–806. (Electronic)). 10.1183/09031936.00185114 25976683

[B20] Jolly M. K. Ward C. Eapen M. S. Myers S. Hallgren O. Levine H. (2018). Epithelial-mesenchymal Transition, a Spectrum of States: Role in Lung Development, Homeostasis, and Disease. Dev. Dyn. 247, 346–358. (Electronic)). 10.1002/dvdy.24541 28646553

[B21] Kapanci Y. Desmouliere A. Pache J. C. Redard M. Gabbiani G. (1995). Cytoskeletal Protein Modulation in Pulmonary Alveolar Myofibroblasts during Idiopathic Pulmonary Fibrosis. Possible Role of Transforming Growth Factor Beta and Tumor Necrosis Factor Alpha. Am. J. Respir. Crit. Care Med. 152, 2163–2169. (Print)). 10.1164/ajrccm.152.6.8520791 8520791

[B22] Khalil N. Bereznay O. Sporn M. Greenberg A. H. (1989). Macrophage Production of Transforming Growth Factor Beta and Fibroblast Collagen Synthesis in Chronic Pulmonary Inflammation. J. Exp. Med. 170, 727–737. (Print)). 10.1084/jem.170.3.727 2475572 PMC2189427

[B23] Khalil N. O'Connor R. Unruh H. W. Warren P. W. Flanders K. C. Kemp A. (1991). Increased Production and Immunohistochemical Localization of Transforming Growth Factor-Beta in Idiopathic Pulmonary Fibrosis. Am. J. Respir. Cel Mol Biol. 5, 155–162. (Print)). 10.1165/ajrcmb/5.2.155 1892646

[B24] Kikkawa T. Sato N. Takahashi G. Aoki K. Hoshikawa K. Akitomi S. (2010). Significance of Measuring S100A12 and sRAGE in the Serum of Sepsis Patients with Postoperative Acute Lung Injury. Dig. Surg. 27, 1421–9883. (Electronic)). 10.1159/000313687 20689292

[B25] Kim D. S. Collard Hr. King T. E. Jr. (2006). Classification and Natural History of the Idiopathic Interstitial Pneumonias. Proc. Am. Thorac. Soc. 3, 285–292. (Print)). 10.1513/pats.200601-005TK 16738191 PMC2658683

[B26] Kim H. J. Perlman D. Tomic R. (2015). Natural History of Idiopathic Pulmonary Fibrosis. Respir. Med. 109, 661–670. (Electronic)). 10.1016/j.rmed.2015.02.002 25727856

[B27] King T. E. Jr. Bradford W. Z. Castro-Bernardini S. Fagan E. A. Glaspole I. Glassberg M. K. (2014). A Phase 3 Trial of Pirfenidone in Patients with Idiopathic Pulmonary Fibrosis. N. Engl. J. Med. 370, 2083–2092. (Electronic)). 10.1056/NEJMoa1402582 24836312

[B28] Lederer D. J. Martinez F. J. (2018). Idiopathic Pulmonary Fibrosis. N. Engl. J. Med. 378, 1811–1823. (Electronic)). 10.1056/NEJMra1705751 29742380

[B29] Lee J. U. Cheong H. S. Shim E. Y. Bae D. J. Chang H. S. Uh S. T. (2017). Gene Profile of Fibroblasts Identify Relation of CCL8 with Idiopathic Pulmonary Fibrosis. BMC Pulm. Med., 1465–993X. (Electronic)). 10.1186/s12931-016-0493-6 PMC5216573 28057004

[B30] Ley B. Collard H. (2013). Epidemiology of Idiopathic Pulmonary Fibrosis. Clin Epidemiol. 5, 483–492. (Print)). 10.2147/CLEP.S54815 24348069 PMC3848422

[B31] Ley B. Collard H. R. King T. E. Jr. (2011). Clinical Course and Prediction of Survival in Idiopathic Pulmonary Fibrosis. Am. J. Respir. Crit. Care Med. 183, 431–440. (Electronic)). 10.1164/rccm.201006-0894CI 20935110

[B32] Li R. Yin Y.-H. Ji X.-L. Liu X. Li J.-P. Qu Y.-Q. (2021). Pan-Cancer Prognostic, Immunity, Stemness, and Anticancer Drug Sensitivity Characterization of N6-Methyladenosine RNA Modification Regulators in Human Cancers. Front. Mol. Biosci. 8, 2296–889X. (Print)). 10.3389/fmolb.2021.644620 PMC8211991 34150845

[B33] Li X. Cai H. Cai Y. Zhang Q. Ding Y. Zhuang Q. (2021). Investigation of a Hypoxia-Immune-Related Microenvironment Gene Signature and Prediction Model for Idiopathic Pulmonary Fibrosis. Front. Immunol. 12, 1664–3224. (Electronic)). 10.3389/fimmu.2021.629854 PMC8236709 34194423

[B34] Lorenz E. Muhlebach M. S. Tessier P. A. Alexis N. E. Duncan Hite R. Seeds M. C. (2008). Different Expression Ratio of S100A8/A9 and S100A12 in Acute and Chronic Lung Diseases. Respir. Med. 102, 567–573. (Print)). 10.1016/j.rmed.2007.11.011 18164192 PMC2347354

[B35] Lu Y. Chen J. Tang K. Wang S. Tian Z. Wang M. (2021). Development and Validation of the Prognostic Index Based on Inflammation-Related Gene Analysis in Idiopathic Pulmonary Fibrosis. Front. Mol. Biosciences 8, 2296–889X. (Print)). 10.3389/fmolb.2021.667459 PMC8339426 34368225

[B36] MacKenzie B. Korfei M. Henneke I. Sibinska Z. Tian X. Hezel S. (2015). Increased FGF1-FGFRc Expression in Idiopathic Pulmonary Fibrosis. Respir. Res. 16, 1465–993X. (Electronic)). 10.1186/s12931-015-0242-2 PMC4495640 26138239

[B37] Martinez F. J. Collard H. R. Pardo A. Raghu G. Richeldi L. Selman M. (2017). Idiopathic Pulmonary Fibrosis. Nat. Rev. Dis. Primers 3, 74. (Electronic)). 10.1038/nrdp.2017.74 29052582

[B38] Mitsi E. Kamng'ona R. Rylance J. Solórzano C. Jesus Reiné J. Mwandumba H. C. (2018). Human Alveolar Macrophages Predominately Express Combined Classical M1 and M2 Surface Markers in Steady State. Respir. Res. 19, 1465–993X. (Electronic)). 10.1186/s12931-018-0777-0 PMC5907303 29669565

[B39] Molgora M. Supino D. Mantovani A. Garlanda C. (2018). Tuning Inflammation and Immunity by the Negative Regulators IL-1R2 and IL-1R8. Immunol. Rev. 281, 233–247. (Electronic)). 10.1111/imr.12609 29247989 PMC5922415

[B40] Mora-Buch R. Dotti I. Planell N. Calderón-Gómez E. Jung P. Masamunt M. C. (2016). Epithelial IL-1R2 Acts as a Homeostatic Regulator during Remission of Ulcerative Colitis. Mucosal Immunol. 9, 950–959. (Electronic)). 10.1038/mi.2015.108 26530134 PMC4917674

[B41] Morbini P. Inghilleri S. Campo I. Oggionni T. Zorzetto M. Luisetti M. (2011). Incomplete Expression of Epithelial-Mesenchymal Transition Markers in Idiopathic Pulmonary Fibrosis. Pathol. - Res. Pract. 207, 559–567. (Electronic)). 10.1016/j.prp.2011.06.006 21798671

[B42] Mubarak K. K. Montes-Worboys A. Regev D. Nasreen N. Mohammed K. A. Faruqi I. (2012). Parenchymal Trafficking of Pleural Mesothelial Cells in Idiopathic Pulmonary Fibrosis. Eur. Respir. J. 39, 133–140. (Electronic)). 10.1183/09031936.00141010 21737551

[B43] Mura M. Porretta M. A. Bargagli E. Sergiacomi G. Zompatori M. Sverzellati N. (2012). Predicting Survival in Newly Diagnosed Idiopathic Pulmonary Fibrosis: a 3-year Prospective Study. Eur. Respir. J. 40, 101–109. (Electronic)). 10.1183/09031936.00106011 22241745

[B44] Newman A. M. Liu C. L. Green M. R. Gentles A. J. Feng W. Xu Y. (2015). Robust Enumeration of Cell Subsets from Tissue Expression Profiles. Nat. Methods 12, 453–457. (Electronic)). 10.1038/nmeth.3337 25822800 PMC4739640

[B45] O’Dwyer D. N. Armstrong M. E. Trujillo G. Fau - Cooke G. Keane M. P. Fallon A. J. (2013). The Toll-like Receptor 3 L412F Polymorphism and Disease Progression in Idiopathic Pulmonary Fibrosis. Am. J. Respir. Crit. Care Med. 188, 1442–1450. (Electronic)). 10.1164/rccm.201304-0760OC 24070541

[B46] Ortiz L. A. Lasky J. Fau - Hamilton R. F. Jr. Holian A. Hoyle G. W. Banks W. (1998). Expression of TNF and the Necessity of TNF Receptors in Bleomycin-Induced Lung Injury in Mice. Exp. Lung Res. 24, 721–743. (Print)). 10.3109/01902149809099592 9839161

[B47] Parker H. S. Leek J. T. Favorov A. V. Considine M. Xia X. Chavan S. (2014). Preserving Biological Heterogeneity with a Permuted Surrogate Variable Analysis for Genomics Batch Correction. Bioinformatics 30, 2757–2763. (Electronic)). 10.1093/bioinformatics/btu375 24907368 PMC4173013

[B48] Piqueras B. Connolly J. Freitas H. Palucka A. K. Banchereau J. (2006). Upon Viral Exposure, Myeloid and Plasmacytoid Dendritic Cells Produce 3 Waves of Distinct Chemokines to Recruit Immune Effectors. Blood. 107, 2613. (Print)). 10.1182/blood-2005-07-2965 16317096 PMC1895384

[B49] Qiu L. Gong G. Wu W. Li N. Li Z. Chen S. (2021). A Novel Prognostic Signature for Idiopathic Pulmonary Fibrosis Based on Five-Immune-Related Genes. Ann. Transl Med. 9, 1570. (Print)). 10.21037/atm-21-4545 34790776 PMC8576669

[B50] Raghu G. Collard H. R. Egan J. J. Martinez F. J. Behr J. Brown K. K. (2011). An Official ATS/ERS/JRS/ALAT Statement: Idiopathic Pulmonary Fibrosis: Evidence-Based Guidelines for Diagnosis and Management. Am. J. Respir. Crit. Care Med. 183, 788–824. (Electronic)). 10.1164/rccm.2009-040GL 21471066 PMC5450933

[B51] Raghu G. Weycker D. Edelsberg J. Bradford W. Z. Oster G. (2006). Incidence and Prevalence of Idiopathic Pulmonary Fibrosis. Am. J. Respir. Crit. Care Med. 174, 810–816. (Print)). 10.1164/rccm.200602-163OC 16809633

[B52] Ragno S. Romano M. Howell S. Pappin D. J. Jenner P. J. Colston M. J. 2001 Changes in Gene Expression in Macrophages Infected with *Mycobacterium tuberculosis*: a Combined Transcriptomic and Proteomic Approach. Immunology 104, 0019–2805. (Print)). 10.1046/j.0019-2805.2001.01274.x PMC1783284 11576227

[B53] Reyfman P. A-O. Walter J. A-O. Joshi N. A-O. Anekalla K. A-O. McQuattie-Pimentel A. C. Chiu S. (2009). Single-Cell Transcriptomic Analysis of Human Lung Provides Insights into the Pathobiology of Pulmonary Fibrosis. Am. J. Respir. Crit. Care Med., 1535–4970. (Electronic)). 10.1164/rccm.201712-2410OC PMC6580683 30554520

[B54] Richards T. J. Kaminski N. Baribaud F. Flavin S. Brodmerkel C. Horowitz D 2012. Peripheral Blood Proteins Predict Mortality in Idiopathic Pulmonary Fibrosis. Am. J. Respir. Crit. Care Med. 185, 1535–4970. (Electronic)). 10.1164/rccm.201101-0058OC PMC3262037 22016448

[B55] Richeldi L. du Bois R. M. Raghu G. Azuma A. Brown K. K. Costabel U. (2014). Efficacy and Safety of Nintedanib in Idiopathic Pulmonary Fibrosis. N. Engl. J. Med. 370, 2071–2082. (Electronic)). 10.1056/NEJMoa1402584 24836310

[B57] Salonen J. A-O. Kreus M. Lehtonen S. Vähänikkilä H. Purokivi M. Kaarteenaho R. Decline in Mast Cell Density during Diffuse Alveolar Damage in Idiopathic Pulmonary Fibrosis. LID 45, 1573–2576. (Electronic)). 10.1007/s10753-021-01582-0 PMC8956519 34686945

[B58] Schupp J. C. Binder H. Jäger B. Cillis G. Zissel G. Müller-Quernheim J. (1932). Macrophage Activation in Acute Exacerbation of Idiopathic Pulmonary Fibrosis. PLoS ONE 10, e0116775–6203. (Electronic)). 10.1371/journal.pone.0116775 PMC4295887 25590613

[B59] Selman M. King T. E. Pardo A. (2001). Idiopathic Pulmonary Fibrosis: Prevailing and Evolving Hypotheses about its Pathogenesis and Implications for Therapy. Ann. Intern. Med. 134, 136–4819. (Print)). 10.7326/0003-4819-134-2-200101160-00015 11177318

[B60] Sgalla G. Iovene B. Calvello M. Ori M. Varone F. Richeldi L. (2018). Idiopathic Pulmonary Fibrosis: Pathogenesis and Management. Respir. Res. 19, 1465–993X. 10.1186/s12931-018-0730-2 PMC5824456 29471816

[B61] Shimbori C. Upagupta C. Bellaye P. S. Ayaub E. A. Sato S. Yanagihara T. (2019). Mechanical Stress-Induced Mast Cell Degranulation Activates TGF-Β1 Signalling Pathway in Pulmonary Fibrosis. BMJ 74, 1468–3296. (Electronic)). 10.1136/thoraxjnl-2018-211516 30808717

[B62] Spagnolo P. Kropski J. A. Jones M. G. Lee J. S. Rossi G. Karampitsakos T. (2021). Idiopathic Pulmonary Fibrosis: Disease Mechanisms and Drug Development. Pharmacol. Ther. 222, 107798. (Electronic)). 10.1016/j.pharmthera.2020.107798 33359599 PMC8142468

[B63] Van Damme J. Proost P. Put W. Arens S. Lenaerts J. P. Conings R. (1994). Induction of Monocyte Chemotactic Proteins MCP-1 and MCP-2 in Human Fibroblasts and Leukocytes by Cytokines and Cytokine Inducers. Chemical Synthesis of MCP-2 and Development of a Specific RIA. J. Immunol. 152, 5495–5502. (Print)). 8189067

[B64] Whyte M. Hubbard R. Meliconi R. Whidborne M. Fau - Eaton V. Bingle C (2000). Increased Risk of Fibrosing Alveolitis Associated with Interleukin-1 Receptor Antagonist and Tumor Necrosis Factor- α Gene Polymorphisms. Am. J. Respir. Crit. Care Med. 162, 755–758. (Print)). 10.1164/ajrccm.162.2.9909053 10934117

[B65] Yao L. Conforti F. Hill C. Bell J. Drawater L. Li J. (2019). Paracrine Signalling during ZEB1-Mediated Epithelial-Mesenchymal Transition Augments Local Myofibroblast Differentiation in Lung Fibrosis. Cell Death Differ 26, 943–957. 10.1038/s41418-018-0175-7 30050057 PMC6252080

[B66] Yoshihara K. Shahmoradgoli M. Martínez E. Vegesna R. Kim H. Torres-Garcia W. (2013). Inferring Tumour Purity and Stromal and Immune Cell Admixture from Expression Data. Nat. Commun. 4, 2041–1723. (Electronic)). 10.1038/ncomms3612 24113773 PMC3826632

[B67] Yu G. Wang L.-G. Han Y. He Q.-Y. (2012). clusterProfiler: an R Package for Comparing Biological Themes Among Gene Clusters. OMICS: A J. Integr. Biol. 16, 284–287. (Electronic)). 10.1089/omi.2011.0118 PMC3339379 22455463

[B68] Zhang L.-M. Zhang Y. Fei C. Zhang J. Wang L. Yi Z.-W. (2019). Neutralization of IL-18 by IL-18 Binding Protein Ameliorates Bleomycin-Induced Pulmonary Fibrosis via Inhibition of Epithelial-Mesenchymal Transition. Biochem. Biophysical Res. Commun. 508, 660–666. (Electronic)). 10.1016/j.bbrc.2018.11.129 30527805

[B69] Zhao J. Zhong A. Friedrich E. E. Jia S. Xie P. Galiano R. D. (2017). S100A12 Induced in the Epidermis by Reduced Hydration Activates Dermal Fibroblasts and Causes Dermal Fibrosis. J. Invest. Dermatol. 137, 1523–1747. (Electronic)). 10.1016/j.jid.2016.10.040 27840235

[B70] Zheng Y. Humphry M. Maguire J. J. Bennett M. R. Clarke M. C. H. (2013). Intracellular Interleukin-1 Receptor 2 Binding Prevents Cleavage and Activity of Interleukin-1α, Controlling Necrosis-Induced Sterile Inflammation. Immunity 38, 285–295. (Electronic)). 10.1016/j.immuni.2013.01.008 23395675 PMC3659285

