# Supplementary figures and images for: Development and Validation of a Novel Gene Signature for Predicting the Prognosis of Idiopathic Pulmonary Fibrosis Based on Three Epithelial-Mesenchymal Transition and Immune-Related Genes
Source: Front Genet. 2022 Apr 26;13:865052. doi: 10.3389/fgene.2022.865052 (PMC9086533; doi:10.3389/fgene.2022.865052)

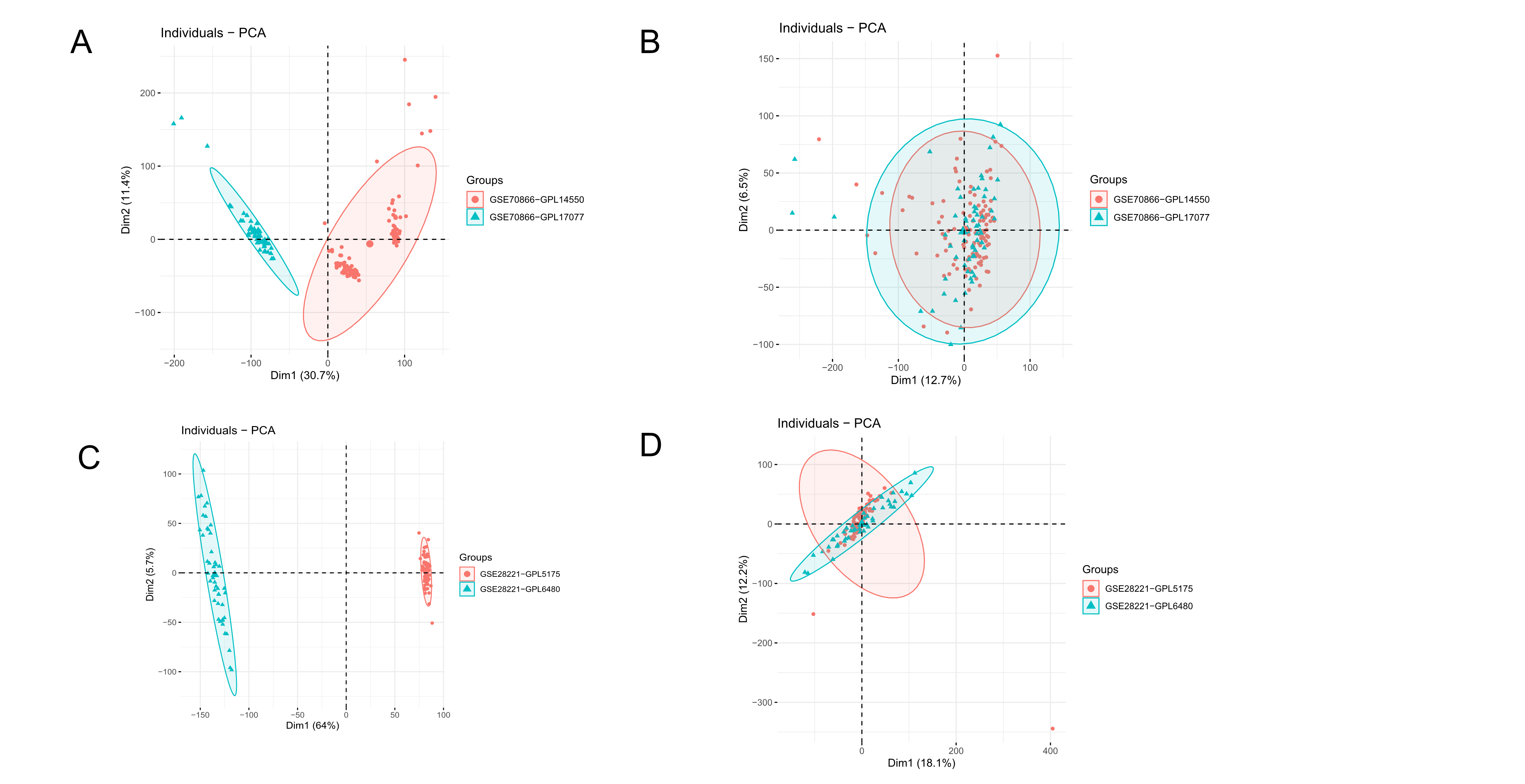

Supplement: Supplementary file 3 [file Image1.TIF]
