# Supplementary material for: Development and Validation of a Novel Gene Signature for Predicting the Prognosis of Idiopathic Pulmonary Fibrosis Based on Three Epithelial-Mesenchymal Transition and Immune-Related Genes
Source: Front Genet. 2022 Apr 26;13:865052. doi: 10.3389/fgene.2022.865052 (PMC9086533; doi:10.3389/fgene.2022.865052)
Supplement: Supplementary file 4 [file Table5.DOCX]

Table S2 These 13 EMT and immune-related genes.

| id | genes |
| --- | --- |
| 1 | DEFA3 |
| 2 | G0S2 |
| 3 | IFITM1 |
| 4 | MMP9 |
| 5 | IL8 |
| 6 | IL1R2 |
| 7 | ADORA2A |
| 8 | S100A12 |
| 9 | PROK2 |
| 10 | CHI3L1 |
| 11 | GNLY |
| 12 | CCL8 |
| 13 | CHST15 |
