# Supplementary material for: Development and Validation of a Novel Gene Signature for Predicting the Prognosis of Idiopathic Pulmonary Fibrosis Based on Three Epithelial-Mesenchymal Transition and Immune-Related Genes
Source: Front Genet. 2022 Apr 26;13:865052. doi: 10.3389/fgene.2022.865052 (PMC9086533; doi:10.3389/fgene.2022.865052)
Supplement: Supplementary file 5 [file Table3.DOCX]

Table 1 The results of multivariate Cox regression analysis.

| id | coef | HR | HR.95L | HR.95H | pvalue |
| --- | --- | --- | --- | --- | --- |
| IL1R2 | 0.177105131 | 1.193756587 | 1.039919913 | 1.370350517 | 0.011867388 |
| S100A12 | 0.18550844 | 1.20383036 | 1.039012538 | 1.394793116 | 0.01353368 |
| CCL8 | 0.12467842 | 1.132784113 | 1.001368767 | 1.281445847 | 0.047512265 |
