# Supplementary material for: Development and Validation of a Novel Gene Signature for Predicting the Prognosis of Idiopathic Pulmonary Fibrosis Based on Three Epithelial-Mesenchymal Transition and Immune-Related Genes
Source: Front Genet. 2022 Apr 26;13:865052. doi: 10.3389/fgene.2022.865052 (PMC9086533; doi:10.3389/fgene.2022.865052)
Supplement: Supplementary file 6 [file Table4.DOCX]

Table S1 Primer information list

| Genes | Primer | size of the amplified product | annealing temperature |
| --- | --- | --- | --- |
| IL1R2 | primers: upstream primer  F: 5'-CTTTCCTGCCGTTCATCTCATAC-3';  downstream primer  R: 5'-CCAAAAGAAGAGAATCCTTGTACCA-3'. | 138bp | 55-60℃ |
| S100A12 | upstream primer  F: 5'-GTTAACATTAGGCTGGGAAGATGAC-3';  downstream primer  R: 5'-CGAACTGAGTATTGGTGGAAGATATTGAC-3'. | 82bp | 55-60℃ |
| CCL8 | upstream primer  F: 5'-GCTCAGCCAGATTCAGTTTCCATTC-3' downstream primer  R: 5'-CTTGCCCCGTTTGGTCTTGA-3'. | 150bp | 55-60℃ |
